# The Level of Knowledge and Attitude Toward Dealing With Fractures at Accident Sites Among Al Baha Population

**DOI:** 10.7759/cureus.69246

**Published:** 2024-09-12

**Authors:** Hasan A AlAidarous, Twfiq A Alghamdi, Hassan S Alomari, Ahmed M Alomari, Ayman M Alzahrani, Waseem A Alghamdi, Fahad J Alzahrani

**Affiliations:** 1 Surgery, Faculty of Medicine, Al Baha University, Al Baha, SAU; 2 Medicine, Faculty of Medicine, Al Baha University, Al Baha, SAU

**Keywords:** emergency, knowledge and awareness, orthopaedics, saudi arabia, trauma

## Abstract

Background: A fractured bone is a medical emergency that causes bone continuity to be partially or completely disrupted. Effective fracture management at accident sites is crucial for minimizing complications and improving outcomes. Despite its importance, this study aimed to assess the level of knowledge and attitude toward fracture management among residents of Al Baha province and explore the association of this knowledge with demographic factors.

Methodology: A cross-sectional descriptive study involving 389 participants was carried out employing a structured online self-administered questionnaire that contained sociodemographic information about the participants along with a basic knowledge assessment regarding fractures at accident sites. To investigate relationships between variables, statistical analysis was used, including the Chi-square test.

Results: This investigation revealed a majority of participants demonstrated moderate to high levels of knowledge about fracture management, with 166 (42.7%) classified as having high knowledge and 187 (48.1%) as having moderate knowledge. Meanwhile, four (1%) were found to have poor levels. Significant associations were found between knowledge levels and gender (p = 0.048), residency (p = 0.014), and marital status (p = 0.011). Males, residents of Al Baha, and married individuals showed the highest levels of knowledge. However, gaps were identified in responses related to open fractures and spine injuries.

Conclusion: In conclusion, a study of 389 participants highlights a generally high level of knowledge about fracture management among Al Baha residents, with notable differences linked to demographic factors. While the findings suggest a solid understanding of basic fracture management principles, there are specific areas where knowledge could be improved. Targeted educational interventions, especially those addressing identified knowledge gaps and tailored to different demographic groups, are recommended to enhance public preparedness and response to fractures.

## Introduction

Before emergency medical assistance arrives, initial care is provided to injured or ill individuals in potentially fatal circumstances in an effort to preserve their lives, prevent more damage, or assist in the healing process [1]. It involves conducting a physical examination and taking immediate actions that can be performed by individuals at the scene of the accident, often with limited medical supplies [2]. Consequently, having a solid foundation of first-aid knowledge is essential. The primary objective prior to reaching a medical facility is to mitigate or reverse potential injuries, including fractures [3].

To commence with, a bone fracture is a medical condition characterized by the partial or complete disruption of the continuity of a bone [4]. It represents the most common form of major trauma injury affecting the human body [5]. According to the World Health Organization (WHO), road traffic accidents account for approximately 1.35 million fatalities worldwide, with an additional 20 to 50 million nonfatal injuries, many of which result in disabilities [6]. This underscores the importance of understanding and effectively managing fractures at accident sites.

Therefore, some previous literature indicates that fractures caused by road traffic incidents constituted 83.4% of all trauma admissions between 1984 and 1989. However, there was no longer evidence of this trend after 1989. Recent studies reveal that injuries to the head, neck, upper, and lower extremities are the most frequently sustained injuries [7]. Moreover, a multinational study conducted in 2023 demonstrated that the average level of basic life support knowledge among non-medical individuals is inadequate [8]. Around the world, road accidents are a major source of health issues and an expense for medical facilities [9]. This highlights the need for improved education and awareness regarding fracture management in the general population.

Despite the critical nature of this issue, as illustrated by a cross-sectional study involving 354 road traffic accident patients with orthopedic injuries, fractures represent the most frequent type of injury, accounting for 71.1% of all injuries, particularly lower limb fractures (42.1%). Orthopedic injuries resulting from traffic accidents are a leading cause of death and disability, with over half of the victims (59.5%) suffering from open wounds [10]. Due to the importance of appropriate fracture management at accident sites, there is a substantial knowledge gap and limited research, particularly in the Al Baha region of the Kingdom of Saudi Arabia. Therefore, this proposed study aims to address these disparities by focusing solely on evaluating the knowledge, attitude, and awareness regarding the management of fractures at accident sites within the Al Baha population.

## Materials and methods

Study design and setting

The study employed a cross-sectional descriptive design to assess the knowledge of fracture management among the residents of Al Baha province. The inclusion criteria encompassed all male and female residents over the age of 18 who consented to participate and were not employed in the healthcare industry. Individuals were excluded if they were not residents of Al Baha, were under the age of 18, declined to participate, or were healthcare providers. After all, the study was conducted after receiving IRB approval no. REC/SUR/BU-FM/2024/76 for May 14, 2024, from the Scientific Research and Ethics Committee of Al Baha University Faculty of Medicine, Saudi Arabia.

Sample size

The sample size was determined using a 95% confidence level and a 5% significance level, calculated with the Raosoft (Raosoft, Inc, Seattle, United States) sample size calculator, resulting in a sample of 384 participants.

Data collection instrument

Data collection was conducted through a structured online self-administered questionnaire, which was distributed to the general population in Al Baha throughout a period from June 2024 to July 2024. The questionnaire was available in both Arabic and English and was designed to assess important aspects related to accident-site fractures. It consisted of two parts: the first part included questions about the participants' socioeconomic background, and the second part included questions on their knowledge and attitude toward fracture management at accident sites. In order to ensure content validity, the questionnaire was meticulously crafted, covering all relevant topics on unintentional fractures, and questions were developed based on a thorough review of the literature and feedback from subject-matter experts.

Statistical analysis

Following the collection of data, the responses were imported into Microsoft Excel (2016; Microsoft Corporation, Redmond, United States) and subsequently transferred to SPSS version 20 (IBM Corporation, Armonk, United States) for statistical analysis. Frequency and percentage were used for the description of categorical variables that were presented as tables and figures.

Scoring system

In addition, to evaluate the gathered information, a scoring system was made to evaluate the participants' knowledge of fracture management at accident sites provided subsequently. The questionnaire included 16 questions, with each correct answer scoring 1 point and each incorrect answer scoring 0 points. Knowledge levels were categorized as follows: high (13-16 points), moderate (9-12 points), low (5-8 points), and poor (less than 4 points). A Chi-square test was used to assess the association between knowledge levels and demographic factors. All statements were considered significant when the p value was lower than 0.05.

## Results

The study included a total of 389 participants. Table 1 shows the sociodemographic information of the participants. The most prevalent age group was 18-25 years, comprising 137 (35.2%) participants, with a majority of males at 219 (56.3%). In terms of residency, most participants were from Al Baha (122, 31.4%) and Al Makhwah (101, 26%). The majority of participants were married (227, 58.4%) and held a university degree (198, 50.9%).

**Table 1:** The sociodemographic factors of the participants

Variables		Count	Percentage	Chi-square, p-value
Gender	Male	219	56.3%	3.5069, 0.0612
	Female	170	43.7%	
Age	18-25	137	35.2%	58.5531, < 0.0001
	26-35	46	11.8%	
	36-45	87	22.4%	
	46-55	97	24.9%	
	> 55	22	5.7%	
Residency	Al Baha	122	31.4%	304.0769, < 0.0001
	Qilwah	38	9.8%	
	Baljurashi	31	8%	
	Al Mandaq	16	4.1%	
	Al Makhwah	101	26%	
	Al Aqiq	19	4.9%	
	Bani Hasan	47	12.1%	
	Al Qura	5	1.3%	
	Ghamid Alzinad	5	1.3%	
	Hajrah	5	1.3%	
Marital status	Single	162	41.6%	6.4888, 0.0109
	Married	227	58.4%	
Educational level	Primary school	1	0.3%	270.6667, < 0.0001
	Intermediate school	5	1.3%	
	High school	99	25.4%	
	Diploma	61	15.7%	
	University	198	50.9%	
	Post-graduated	25	6.4%	

The basic knowledge and proper actions are assessed through questions as presented in Table 2. The study's knowledge assessment revealed that the majority of participants identified motor vehicle accidents as the most common cause of bone fractures at accident scenes (270, 69.4%). When encountering a person with suspected fractures, 300 (77.1%) correctly stated that contacting emergency services is the first step. The common sign associated with fractures was correctly identified as swelling by 309 (79.4%) participants. An open fracture was correctly described as when the bone protrudes out from the skin (265, 68.1%) of respondents. To stabilize a suspected fracture in the upper arm, 203 (52.2%) participants correctly recommended using slings for fixation. A significant proportion (352, 90.5%) of participants correctly indicated that it is not possible to realign a fracture manually without proper medical training. When dealing with fractures, 262 (67.4%) knew to avoid moving an injured person unnecessarily. Moreover, a sterile gauze or a clean cloth was correctly identified as the appropriate material to cover an open fracture wound by 270 (69.4%) participants. In terms of emergency situations involving suspected neck or spine fractures, 285 (73.3%) participants correctly suggested waiting for medical professionals before taking any action. Along with keeping the injured person calm and reassured while seeking medical help, this was correctly recognized as important by 382 (98.2%) respondents. The purpose of applying a cold compress to the fracture site was correctly identified as reducing pain and swelling by 329 (84.6%) participants. When it comes to a complete loss of sensation in the affected area after an accident, it was correctly indicated as a sign of a potentially serious fracture by 290 (74.6%) respondents. When providing first aid for a suspected fracture, 321 (82.5%) participants correctly emphasized keeping the affected limb still and immobile. Additionally, 337 (86.6%) respondents agreed that fractures are not always visible to the naked eye. To minimize the risk of infection when dealing with an open fracture, 197 (50.6%) correctly recommended all appropriate measures, including cleaning the wound with water, applying antibiotic ointment, and covering the wound with a sterile dressing. Lastly, if a fracture is suspected in an unconscious, non-breathing victim, 230 (59.1%) correctly advised starting cardiopulmonary resuscitation (CPR) immediately.

**Table 2:** Responses considering general knowledge and attitudes *Correct answer. CPR: cardiopulmonary resuscitation.

Questions	Answers	Count	Percentage
What is the most common cause of bone fractures at accident sites?	Falls	94	24.2%
	Motor vehicle accidents*	270	69.4%
	Sport injuries	16	4.1%
	Workplace accidents	9	2.3%
What is the first step you should take when encountering a person with a suspected fracture at an accident site?	Emergency contact*	300	77.1%
	Move the person away from the accident site	40	10.3%
	Giving pain relief medication	6	1.5%
	Try to stabilize the fracture immediately	43	11.1%
Which of the following signs is commonly associated with a fracture?	Nausea	45	11.6%
	Dizziness	24	6.2%
	Swelling*	309	79.4%
	Fatigue	11	2.8%
Which of the following examples is an open fracture?	A fracture in which a bone breaks into two separate pieces	56	14.4%
	A fracture occurs when the bone protrudes out of the skin*	265	68.1%
	A fracture in which the bone cracks but remains within the skin	20	5.1%
	A fracture where the bone is dislocated from its normal position	48	12.3%
What is the correct way to stabilize a suspected fracture in the upper arm?	Apply a sling for fixation*	203	52.2%
	Use of splint	156	40.1%
	Apply a cold compress	25	6.4%
	Perform gentle massage at the area	5	1.3%
True or False: It is safe to attempt to realign a fracture manually without proper medical training.	True	37	9.5%
	False*	352	90.5%
Which of the following actions should be avoided when dealing with fractures at the scene of an accident?	Moving the injured person unnecessarily*	262	67.4%
	Applying direct pressure to the fracture site	56	14.4%
	Immobilization of the affected limb	48	12.3%
	Providing psychological and emotional support	23	5.9%
What should be used to cover an open fracture wound?	Sterile gauze or a clean cloth*	270	69.4%
	Adhesive tape	19	4.9%
	Tourniquet	19	4.9%
	Nothing, leave the wound uncovered	81	20.8%
What should you do if you suspect a fracture in the neck or spine?	Place a cervical collar	74	19%
	Move the person to a comfortable position	22	5.7%
	Perform CPR immediately	8	2.1%
	Wait until medical professionals arrive before taking any action*	285	73.3%
True or False: It is essential to keep the injured person calm and reassured while waiting for medical help.	True*	382	98.2%
	False	7	1.8%
What is the purpose of applying a cold compress to the fracture site?	To reduce pain and swelling*	329	84.6%
	To promote blood circulation	41	10.5%
	To accelerate bone healing	10	2.6%
	To prevent infection	9	2.3%
Which of the following is a sign of a potentially serious fracture?	Mild pain and discomfort	26	6.7%
	Complete loss of sensation in the injured area*	290	74.6%
	Temporary discoloration of the skin	49	12.6%
	Tingling sensation in the fingers or toes	24	6.2%
When providing first aid for a suspected fracture, it is important to:	Apply pressure directly to the fracture site	39	10%
	Attempt to move the fractured bone back into place	21	5.4%
	Keep the injured limb still and immobile*	321	82.5%
	Encouraging the injured person to walk on the fractured limb	8	2.1%
True or False: Fractures are always visible to the naked eye.	True	52	13.4%
	False*	337	86.6%
Which of the following actions should be taken to minimize the risk of infection when dealing with an open fracture?	Clean the wound with water	15	3.9%
	Apply an antibiotic ointment to the wound	48	12.3%
	Cover the wound with a sterile dressing	129	33.2%
	All of the above*	197	50.6%
What should you do if you suspect a fracture but the victim is unconscious and not breathing?	Begin CPR immediately*	230	59.1%
	Apply pressure to the fracture site	7	1.8%
	Wait until medical professionals to arrive before taking any action	99	25.4%
	I don't know	53	13.6%

**Figure 1:**
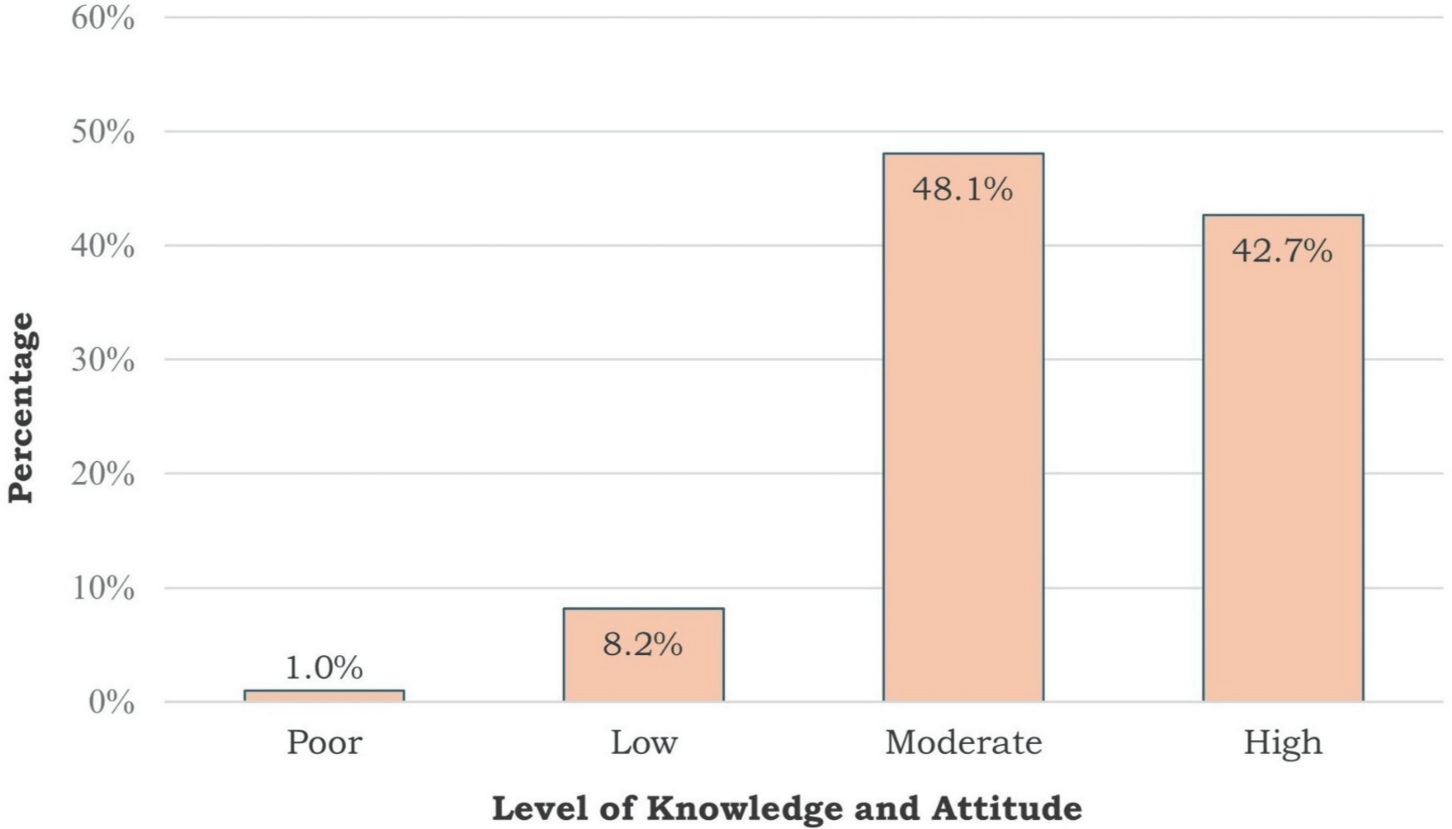
The participants' level of knowledge

In addition, Figure 1 illustrates how the participants were categorized according to their attitude levels. The level of knowledge among the participants varied, with 166 (42.7%) having a high level of knowledge and 187 (48.1%) having a moderate level. Meanwhile, 32 (8.2%) had a low level, and finally, four (1.0%) had a poor level.

The association between the level of knowledge and various sociodemographic factors is illustrated in Table 3. Gender was significantly associated with knowledge levels, as males had a higher proportion of high knowledge (101, 46.1%) compared to females (65, 38.2%) (p = 0.048). Residency also showed a significant association, with participants from Al Baha having the highest proportion of moderate knowledge (64, 52.5%) and those from Al Qura having the highest proportion of high knowledge (4, 80%) (p = 0.014). Marital status was another significant factor, with married participants showing a higher proportion of moderate knowledge (121, 53.3%) compared to single participants (66, 40.7%) (p = 0.011). Although not statistically significant, trends were observed in educational levels, with post-graduates having the highest proportion of high knowledge (16, 64%) and high school graduates showing a more evenly distributed knowledge level (p = 0.051). Age was not significantly associated with knowledge levels, although participants over 55 showed a tendency toward high knowledge (12, 54.5%) (p = 0.115).

**Table 3:** The association between level of knowledge and sociodemographic factors

Variables		Knowledge Level								Chi-square, p-value
		Poor		Low		Moderate		High		
		Count	Percentage	Count	Percentage	Count	Percentage	Count	Percentage	
Gender	Male	2	0.9%	23	10.5%	93	42.5%	101	46.1%	13.56, 0.004
	Female	2	1.2%	9	5.3%	94	55.3%	65	38.2%	
Age	18-25	2	1.5%	19	13.9%	55	40.1%	61	44.5%	27.91, 0.006
	26-35	1	2.2%	5	10.9%	21	45.7%	19	41.3%	
	36-45	0	0%	5	5.7%	47	54%	35	40.2%	
	46-55	1	1%	3	3.1%	54	55.7%	39	40.2%	
	> 55	0	0%	0	0%	10	45.5%	12	54.5%	
Residency	Al Baha	3	2.5%	11	9%	64	52.5%	44	36.1%	43.41, 0.001
	Qilwah	1	2.6%	2	5.3%	16	42.1%	19	50%	
	Baljurashi	0	0%	3	9.7%	19	61.3%	9	29%	
	Al Mandaq	0	0%	1	6.3%	7	43.8%	8	50%	
	Al Makhwah	0	0%	3	3%	49	48.5%	49	48.5%	
	Al Aqiq	0	0%	6	31.6%	4	21.1%	9	47.4%	
	Bani Hasan	0	0%	3	6.4%	22	46.8%	22	46.8%	
	Al Qura	0	0%	1	20%	0	0%	4	80%	
	Ghamid Alzinad	0	0%	2	40%	2	40%	1	20%	
	Hajrah	0	0%	0	0%	4	80%	1	20%	
Marital status	Single	2	1.2%	21	13%	66	40.7%	73	45.1%	15.09, 0.002
	Married	2	0.9%	11	4.8%	121	53.3%	93	41%	
Educational level	Primary school	0	0%	0	0%	0	0%	1	100%	28.22, 0.005
	Intermediate school	0	0%	0	0%	3	60%	2	40%	
	High school	2	2%	17	17.2%	38	38.4%	42	42.4%	
	Diploma	0	0%	5	8.2%	32	52.5%	24	39.3%	
	University	2	1%	9	4.5%	106	53.5%	81	40.9%	
	Post-graduated	0	0%	1	4%	8	32%	16	64%	

## Discussion

At the scene of an accident, efficient fracture intervention is essential to reduce harm and improve outcomes. This study aimed to assess the level of knowledge about fracture management at accident sites among the residents of Al Baha province and its associations with various demographic factors. Among 489 participants, the findings revealed varying levels of knowledge, with significant differences associated with gender, residency, and marital status.

The majority of participants exhibited moderate to high levels of knowledge about fracture management. Specifically, 166 (42.7%) had higher knowledge, and 187 (48.1%) had moderate knowledge. This reflects a generally good level of awareness among the participants. However, there is still a significant portion with low or poor knowledge, combined with 36 (9.2%), indicating room for improvement in public education on this critical health issue. This provides valuable insights into participants' knowledge about fracture management at accident sites. The high proportion of correct responses to questions such as the common cause of fractures and the appropriate initial steps in fracture management suggests a solid baseline understanding among the participants. For instance, the majority correctly identified motor vehicle accidents as a leading cause of fractures (270, 69.4%) and recognized that emergency contact should be the first action when encountering suspected fractures (300, 77.1%).

Many previous studies in the literature showed that vehicle accidents are the leading cause of fracture globally [3,10-12]. This level of awareness aligns with previous research indicating that public education in first aid and emergency responses can significantly impact knowledge and preparedness [13,14]. However, the data also reveal gaps in knowledge, particularly concerning the management of open fractures and the application of first aid. Most participants correctly identified an open fracture as one where the bone protrudes out from the skin (265, 68.1%). Meanwhile, there was variability in responses about stabilizing fractures and dealing with suspected neck or spine injuries. This variability underscores the need for more targeted educational interventions to address these specific areas. In addition, previous studies have highlighted the importance of detailed training in fracture management to ensure that individuals can perform accurate and effective first aid in emergency situations [15]. Enhancing public education in these areas could improve the overall effectiveness of first aid responses and potentially reduce complications associated with fractures.

Gender was significantly associated with knowledge levels, with males showing a higher proportion of high knowledge (101, 46.1%) compared to females (65, 38.2%). This disparity could be attributed to several factors. Men may have more exposure to environments where accidents and injuries are more common, such as workplaces or during transportation [16]. Additionally, cultural factors in Saudi Arabia might influence the educational opportunities and experiences available to men and women differently, potentially affecting their knowledge levels [17]. Therefore, previous studies have highlighted gender differences in health literacy, with men often having more opportunities to engage in activities that could enhance their practical health knowledge [18,19]. This trend is consistent with the findings of this study and suggests that targeted educational interventions might be necessary to bridge this knowledge gap between genders. Marital status, on the other hand, has also shown a significant association with knowledge levels. Married participants demonstrated a higher proportion of knowledge (93, 41%) compared to single participants (73, 45.1%). This finding could be related to the responsibilities and experiences that come with marriage and family life, potentially leading to greater exposure to health-related information and practices. Married individuals often engage in more responsibilities of caring for a family, which might necessitate a greater understanding of basic first aid and injury management [20]. These findings underscore the importance of considering marital status when designing educational interventions, as it can influence how individuals acquire and utilize health knowledge.

Moreover, educational level trends indicate that higher education levels were associated with better knowledge of fracture management. Post-graduates had a high level of knowledge (16, 64%), compared to high school graduates (42, 42,4%). This trend aligns with existing literature suggesting that higher education correlates with better health literacy [21,22]. It should be noted that occasionally an educational degree cannot contribute to or determine the level of attitude in dealing with fractures at accident sites. This is based on this investigation, which showed that individuals with a primary school degree (1, 100%) can have an excellent attitude. This can be due to other contributing factors such as experimental, social, or environmental impact. But still, education provides individuals with critical thinking skills, access to information, and opportunities to engage in health-related learning. Therefore, individuals with higher education levels are more likely to have better health knowledge and practices. This finding highlights the importance of integrating health education into the formal education system to ensure that individuals acquire essential health knowledge early in life. Apart from this, age was not significantly associated with knowledge levels, although participants over 55 showed a tendency toward high knowledge (12, 54.5%). This trend could be due to the accumulation of life experiences and exposure to health-related issues over time. Older adults often have more encounters with the healthcare system, either personally or through caring for others, which can enhance their knowledge and understanding of health management [23,24].

Ultimately, the findings of this study have several implications for public health initiatives in Al Baha province. First, there is a need for targeted educational programs to address the knowledge gaps identified, particularly among females, residents of certain regions, and individuals with lower educational levels. Public health campaigns should be designed to be culturally sensitive and accessible to all demographic groups. Second, the significant associations between knowledge levels and demographic factors suggest that a one-size-fits-all approach may not be effective. Instead, tailored interventions that consider the specific needs and contexts of different demographic groups are necessary. For example, community-based programs in rural areas, school-based health education, and targeted campaigns for women could help improve knowledge levels more effectively. Third, the role of social determinants of health, such as education and marital status, should be recognized and addressed in public health planning. Ensuring equitable access to education and healthcare resources can help reduce disparities in health knowledge and outcomes.

## Conclusions

In conclusion, an investigation of 389 participants highlights varying levels of knowledge about fracture management among the residents of Al Baha province and the significant associations with gender, residency, and marital status. While the overall knowledge levels are good, there are clear areas for improvement. Tailored public health interventions that consider the specific needs of different demographic groups are essential to enhancing fracture management knowledge and improving health outcomes. By addressing these knowledge gaps, we can ensure better preparedness and response to accidents and injuries, ultimately contributing to the overall health and well-being of the community.
